# Interaction analysis of lipid accumulation product and family history of diabetes on impaired fasting glucose and diabetes risk in population with normotension in Eastern China: a community-based cross-sectional survey

**DOI:** 10.1186/s13690-022-00972-6

**Published:** 2022-10-01

**Authors:** Li Shu, Yingying Zhao, Yanqi Shen, Linlin Jia, Jiaye Zhang

**Affiliations:** 1 School of Public Health, Bengbu Medical College, Bengbu, Anhui Province China; 2 Bengbu Health Board, Bengbu, Anhui Province, China

**Keywords:** Interaction, LAP, IFG, Diabetes, Normotension

## Abstract

**Background:**

Lipid accumulation product (LAP) is considered to be a new convenient useful indicator to assess the visceral fat. Therefore, we aimed to evaluate the risk factors of impaired fasting glucose (IFG) and diabetes, and explore the possible interacting influences of LAP with other factors on the risk of IFG and diabetes among Chinese normotension adults.

**Methods:**

A multistage stratified cluster sampling method was conducted to select urban residents in Bengbu, China. For each eligible participant, data on questionnaire survey, anthropometric measurements and laboratory tests were obtained. The effects of body mass index (BMI), waist circumference (WC), waist to height ratio (WHtR) and LAP for predicting IFG and diabetes were performed by multiple logistic regressions and receiver operating characteristic (ROC) analyses. The interaction effects were evaluated by relative excess risk of interaction (RERI), attributable proportion due to interaction (AP) and synergy index (SI).

**Results:**

Six thousand, four hundred sixty-seven normotension subjects (2695 men and 3772 women) were enrolled in our study, the prevalence of IFG and diabetes were 9.37% and 14.33%, respectively. When assessed using ROC curve analysis, LAP exhibited higher diagnostic accuracy for identifying IFG and diabetes than BMI, the area under the AUC curve was 0.650 (95% CI: 0.637 to 0.662). After adjustment for age, sex, educational level and other confounding factors, multivariate logistic regression analyses indicated that subjects with the fourth quartile of LAP were more likely to develop IFG (adjusted OR: 2.735, 95% CI: 1.794–4.170) and diabetes (adjusted OR: 1.815, 95% CI: 1.297–2.541) than those with the first quartile. A significant interaction between LAP and family history of diabetes was observed in participants (RERI = 1.538, 95%CI: 0.167 to 3.612; AP = 0.375, 95%CI: 0.118 to 0.631; SI = 1.980, 95%CI: 1.206 to 3.251). However, a significant interaction between LAP and abdominal obesity was indicated by the value of RERI (1.492, 95%CI: 0.087 to 3.723) and AP (0.413, 95%CI: 0.014 to 0.756), but not the value of SI (1.824, 95%CI: 0.873 to 3.526).

**Conclusion:**

Our results demonstrated that there might be synergistic effect between LAP and family history of diabetes on the risk of IFG and diabetes.

**Supplementary Information:**

The online version contains supplementary material available at 10.1186/s13690-022-00972-6.

## Background

Type 2 diabetes is considered to be one of the most prevalent noncommunicable disease worldwide, the number of adults with diabetes worldwide was estimated to be 366 million in 2011 and will reach 552 million by 2030 [1, 2]. The etiology of diabetes is not well defined, it is assumed to be a polygenic disorder involving interactions between environmental and genetic risk factors [3, 4]. Meanwhile, most patients often undiagnosed for many years while the patient progresses symptom-free through the earlier stage of diabetes known as impaired fasting glucose (IFG), IFG is a manifestation of glucose metabolism disorder in the early period [5]. A report in China demonstrated that nearly 148.2 million adults were suffering from IFG [6], it is more likely for IFG patients to further progress to diabetes. A large amount evidence showed that IFG and diabetes were closely related to cardiovascular events [7–9], a cohort study carried out in rural areas of China showed that the cumulative incidence of hypertension was substantially higher in IFG and diabetes groups while compared with normal blood glucose group [10]. Hence, it is necessary to search the risk factors and analyze how synergisms between them to influence the development of IFG and diabetes.

Body mass index (BMI) is the recommended indicator to assess and diagnose obesity [11, 12], and in assessement related in adults with diabetes [13], but there is increasing evidence indicating that BMI is not the ideal body fat index [14], it has limitation to differentiate fat and muscle mass and can not reflect central obesity. A research conducted in America showed that 33% of elderly adults at a normal weight defined by BMI have pre-diabetes [15]. Schulze et al. [16] reported that WC and WHtR instead of BMI considering their positive correlations with IFG and diabetes, however, WC and WHtR did not show the excess body fat in circulating blood [17]. However, it is not clear whether BMI can affect diabetes, one study conducted among U.S. elderly from 1991–2010 showed that elevated BMI is associated with increased risk of developing diabetes [18]. Additionally, in another population-based cohort study, BMI was not association with diabetes [13]. Therefore, it is urgent to explore other valuable diabetes predicators to enhance screening accuracy.

The lipid accumulation product (LAP) is a powerful parameter based on WC and serum triglycerides (TG) for predicting diabetes in adults by Kahn et al. [19, 20]. It is an emerging indicator that indirectly expresses central fat accumulation and has been developed to be independently associated with impaired fasting glucose, type 2 diabetes, coronary heart disease and metabolic syndrome [21–23]. Several population-based studies have emphasized the association between LAP and metabolic diseases in different countries [24–26], but there are few studies to investigate the association between LAP and diabetes in China. Anhui, a developing province in the center of China, has experienced rapid economic development. At the same time, its residents have experienced huge lifestyle changes, such as high-fat diet, reduced physical activity and long-term sedentary work, all of which are regarded as major risk factors for diabetes, thus, the applicability of LAP in predicting IFG and diabetes in Anhui province is worth studying.

In the last few decades, it has been established that people with a family history of diabetes are at a high risk for developing diabetes [27, 28], however, there were disagreements on whether or not family history has a significant impact on diabetes progress [29, 30]. A family history of diabetes is considered to be an indicator of genetic susceptibility, and people with a family history of diabetes have a significantly higher risk than others. In addition, as IFG and diabetes are polygenic inheritance patterns, the joint influence of risk factors may aggravate the disease [31], but there are few studies on the interaction between family history of diabetes and other risk factors. Meanwhile, hypertension is also a relatively independent risk factor for diabetes [32], so we performed a cross-sectional study in a large-scale population without hypertension patients to evaluate the interaction effects between family history of diabetes and LAP on IFG and diabetes, and to provide new initiatives for the prevention of IFG and diabetes.

In this study, we aimed to (1) analyzing the association between LAP and risk of IFG and diabetes in Bengbu city, (2) comparing the predict abilities of BMI, WC, WHtR and LAP, (3) assessing the possible interactive effects between LAP and family history on IFG and diabetes risk.

## Methods

### Study population

A cross-sectional survey based on community health management was conducted in the Bengbu city of Anhui province in center China, from July to November 2018. The study participants whose age ≥ 45 years old were selected by a multistage and stratified random sampling method from eleven communities. Residents were selected by community health centers who lived in the selected communities for at least 6 months and willing to cooperate with this project. The following exclusion criteria were as applied: 1) had no ability to communicate with investigators; 2) disagreed to participate in this survey; 3) did not complete the whole survey. A total of 9477 individuals were enrolled in our study, 338 participants who lacked demographic data, anthropometric tests and laboratory examinations were excluded. Meanwhile, 2672 hypertension residents were excluded to select for the normotension individuals. Finally, 6467 residents who had complete data were included in our analysis. The included individuals and excluded individuals had no statistically significant differences in age and sex. Informed written consent was obtained from all participants. The study was supported by Bengbu Health Board and approved by the Ethics Committee of Bengbu Medical College. The procedure of this survey is in accordance with the principles of the 1983 Declaration of Helsinki.

### Questionnaire survey

All subjects were interviewed by trained interviewers by a self-designed questionnaire to collect information on age, sex, educational level, marital status, smoking, drinking, family history of diabetes, physical activity. Educational level was categorized as “middle school graduate or lower” and “high school graduate or higher”. Marital status was classified as “currently married” and “currently not married”. Smoking and drinking were defined as “never”, “current (smoking or drinking regularly in the recent half year)” or “ever (quit smoking or drinking for more than half a year)”. For family history, we defined it as one parent or both parents having diabetes. Physical activity was classified as “sufficient activity (exercised more than 150 min/ week)” and “insufficient activity (exercised less than 150 min/ week)” [33].

### Anthropometric tests and laboratory examinations

Height and weight were measured with the participants to take off their shoes and wear lightweight clothing by trained investigators using calibrated weighing scales. WC was measured at the level of 1 cm above the navel using an un-stretched tape measure. Systolic blood pressure (SBP) and diastolic blood pressure (DBP) were measured with participants in a sitting position, using a corrected mercury sphygmomanometer in the right arm. Three measurements of SBP and DBP were required for each subject after a 10 min rest, and the subject’s BP was calculated as the average of the three measurements.

Blood samples were collected in the morning after an overnight fasting for more than 8 h from the subjects, the serum was isolated within 2 h. The measurement of fasting plasma glucose (FPG), total cholesterol (TC) and triglycerides (TG) was performed using an automatic biochemical analyzer (Modular P800, Switzerland).

### Definitions

1. BMI was calculated as weight in kilograms divided by the square of height in meters, BMI ≥ 28 was defined as general obesity [34].
2. Abdominal obesity was defined as WC ≥ 90 cm for men and WC ≥ 85 cm for women [35].
3. WHtR was calculated as WC (cm) divided by the height (cm) [36].
4. Hypertension was defined as SBP ≥ 140 mmHg and/or DBP ≥ 90 mmHg and/or with a history of antihypertensive medications [37].
5. Diabetes was diagnosed as FPG ≥ 7.0 mmol/L and/or use of hypoglycemic drugs within 2 weeks of enrollment. IFG was defined as 6.1 mmol/L ≤ FPG < 7.0 mmol/L [38].
6. Hypercholesteremia was diagnosed as TC ≥ 5.72 mmol/L. Hypertriglyceridemia was diagnosed as TG ≥ 1.70 mmol/L [39].
7. LAP was calculated as [WC (cm) -65] × [TG (mmol/L)] for males and [WC (cm) -58] × [TG (mmol/L)] for females [20].

### Statistics

The data was organized in the EpiData 3.1 software using double entry approach, an all statistical analyses were performed with IBM SPSS Statistics ver. 22.0 (IBM Co., Armonk, NY, USA) software. Firstly, data was presented as the mean ± standard deviation for continuous variable and percentage for categorical variable, the differences in continuous or categorical variable between NFG, IFG and diabetes groups were compared by One-way ANOVA test or Chi-squared test. Furthermore, LAP was divided into four groups (Q_1_, Q_2_, Q_3_ and Q_4_) by quartiles, multivariate logistic regression was applied to evaluate the risk factors for IFG and diabetes, followed by the calculation of odds ratio (OR) with corresponding 95% confidence interval (95% CI). Thirdly, the receiver operating characteristic (ROC) analysis was determined by MedCalc Version 18 (DEMO) (MedCalc Software bvba, Ostend, Belgiumm) software, to identify the superior obesity index and the best cut-off value of LAP to predict IFG and diabetes risk. The area under the ROC curves (AUC) of BMI, WC, WHtR and LAP were calculated, and then compared by non-parametric significance test (statistics of *Z*). Finally, the interaction effects were evaluated by relative excess risk of interaction (RERI = OR_11_-OR_10_-OR_01_ + 1), attributable proportion due to interaction (AP = [OR_11_- OR_10_-OR_01_ + 1]/OR_11_) and synergy index (SI = [OR_11_-1]/[OR_01_-1] + [OR_10_-1]). The excel table designed by Andersson et al. were used to calculate all of these indicators [33, 40]. If the 95% CI of RERI and AP do not include 0, the 95% CI of SI do not include 1, the interactions are statistically significant. The *P*-value < 0.05 (two-tailed) was set for statistical significance.

## Results

Table 1 shows the demographic characteristics of the 6467 participants (2695 men and 3772 women) with normotension stratified by their FPG levels. The total prevalence of NFG, IFG and diabetes was 76.30%, 9.37% and 14.33%, respectively. The mean age for NFG, IFG and diabetes groups were 62.56 ± 6.06, 62.72 ± 5.97 and 62.47 ± 5.74 years old, respectively. Significant difference was presented in family history of diabetes (*p* < 0.001) between NFG, IFG and diabetes groups. Anthropometric measurements found significant differences in BMI (*p* < 0.001), WC (*p* < 0.001), WHtR (*p* < 0.001), LAP (*p* < 0.001), general obesity (*p* < 0.001) and abdominal obesity (*p* < 0.001) between the groups. Laboratory examinations found significant differences in systolic blood pressure (*p* < 0.001), fasting plasma glucose (*p* < 0.001) and triglyceride (*p* < 0.001) between the groups.

**Table 1:** Participants characteristics (mean, standard deviation, number, frenquency and *p*-value) of the study population in country (Bengbu city, Anhui province, China), 2018

Variables	NFG (*N* = 4934)	IFG (*N* = 606)	Diabetes (*N* = 927)	*F/χ*^*2*^	*P*-value
Age (years)	62.56 ± 6.06	62.72 ± 5.97	62.47 ± 5.74	0.327	0.721
Sex [n(%)]					
Male	2043(41.4)	247(40.8)	405(43.7)	1.903	0.386
Female	2891(58.6)	359(59.2)	522(56.3)		
Education level [n(%)]					
Middle school graduate or lower	4259(86.3)	527(87.0)	797(86.0)	0.305	0.859
High school graduate or higher	675(13.7)	79(13.0)	130(14.0)		
Marital status [n(%)]					
Currently married	4354(88.2)	536(88.4)	815(87.9)	0.115	0.944
Currently not married	580(11.8)	70(11.6)	112(12.1)		
Smoking [n(%)]	404(8.2)	48(7.9)	79(8.5)	0.190	0.909
Drinking [n(%)]	521(10.6)	57(9.4)	82(8.8)	2.967	0.227
BMI (kg/m^2^)	24.21 ± 3.14	25.18 ± 3.22	25.22 ± 3.29	56.545	**0.000**
WC (cm)	84.63 ± 9.30	87.08 ± 9.76	87.61 ± 9.82	51.054	**0.000**
WHtR	0.53 ± 0.06	0.54 ± 0.06	0.54 ± 0.06	39.272	**0.000**
General obesity [n(%)]	579(11.7)	104(17.2)	165(17.8)	34.812	**0.000**
Abdominal obesity [n(%)]	1887(38.2)	302(49.8)	477(51.5)	76.675	**0.000**
Systolic blood pressure (mmHg)	124.98 ± 9.33	127.16 ± 8.21	126.64 ± 8.63	24.969	**0.000**
Diastolic blood pressure (mmHg)	76.06 ± 7.28	76.62 ± 6.96	76.26 ± 7.27	1.795	0.166
Fasting plasma glucose (mmol/L)	4.94 ± 0.56	6.49 ± 0.27	9.25 ± 2.35	7187.339	**0.000**
Total cholesterol (mmol/L)	4.88 ± 1.15	4.97 ± 1.08	4.90 ± 1.29	1.877	0.153
Triglyceride (mmol/L)	1.44 ± 1.03	1.63 ± 1.12	1.80 ± 1.43	46.717	**0.000**
Family history of diabetes [n(%)]	708(14.3)	142(23.4)	268(28.9)	133.369	**0.000**
LAP	35.17 ± 31.49	43.68 ± 38.94	48.83 ± 44.79	70.659	**0.000**

**Fig. 1:**
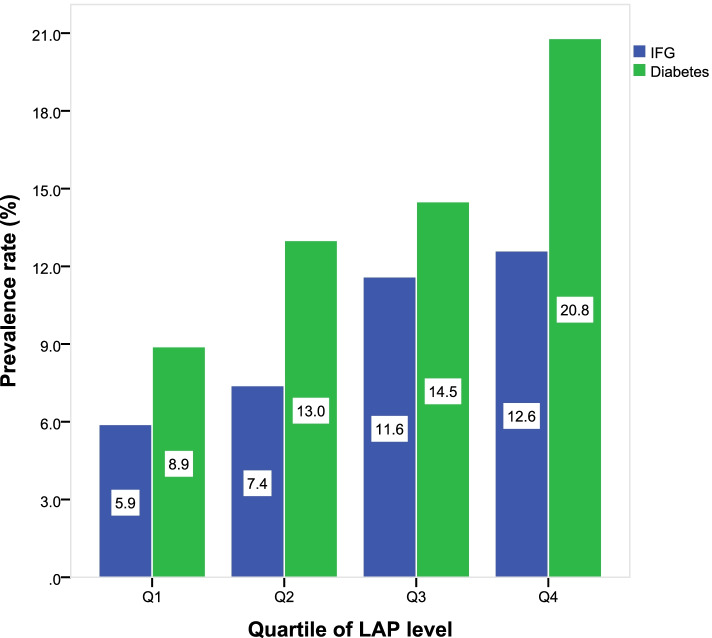
Prevalence of impaired fasting glucose and diabetes in different lipid accumulation product quartiles of the study population in country (Bengbu city, Anhui province, China), 2018 (*P* for trend < 0.001 for impaired fasting glucose and diabetes groups) (Q_1_: < 18.17; Q_2_:18.17 to 28.79; Q_3_: 28.80 to 46.19; Q_4_: ≥ 46.20)

As for LAP, we divided it into four groups by quartile to investigate the relationship between LAP and cardiovascular risk factors, the results showed that variables such as BMI (*p* < 0.001), WC (*p* < 0.001), WHtR (*p* < 0.001), FPG (*p* < 0.001), TC (*p* < 0.001), TG (*p* < 0.001) and so on were relatively elevated in participants with higher LAP quartiles (Table 2). As shown in Fig. 1, the prevalence of IFG and diabetes gradually increased across LAP quartiles (*P* for trend < 0.001 for both groups).

**Table 2:** Comparisons of cardiovascular risk factors among four quartiles of lipid accumulation product of the study population in country (Bengbu city, Anhui province, China), 2018

Variables	LAP				*F/χ*^*2*^	*P*-value
	Q_1_ (< 18.17)	Q_2_ (18.17–28.79)	Q_3_ (28.80–46.19)	Q_4_ (≥ 46.20)		
n	1616	1601	1622	1628	-	-
Age (years)	63.28 ± 6.41	62.57 ± 6.05	62.40 ± 5.89	62.03 ± 5.58	12.480	0.000
Sex [n(%)]						
Male	918(56.8)	680(42.5)	608(37.5)	489(30.0)	255.088	0.000
Female	698(43.2)	921(57.5)	1014(62.5)	1139(70.0)		
Smoking [n(%)]	170(10.5)	111(6.9)	123(7.6)	127(7.8)	16.110	0.001
Drinking [n(%)]	215(13.3)	154(9.6)	164(10.1)	127(7.8)	27.823	0.000
BMI (kg/m^2^)	22.38 ± 2.72	23.96 ± 2.71	25.09 ± 2.84	26.34 ± 3.09	568.279	0.000
WC (cm)	77.91 ± 6.20	83.38 ± 7.18	87.41 ± 7.78	92.38 ± 9.83	985.462	0.000
WHtR	0.48 ± 0.04	0.52 ± 0.04	0.54 ± 0.05	0.58 ± 0.06	1096.950	0.000
General obesity [n(%)]	46(2.8)	119(7.4)	238(14.7)	445(27.3)	487.286	0.000
Abdominal obesity [n(%)]	103(6.4)	464(29.0)	858(52.9)	1241(76.2)	1823.565	0.000
SBP (mmHg)	124.05 ± 9.79	125.38 ± 9.17	125.99 ± 8.59	126.27 ± 8.92	18.890	0.000
DBP (mmHg)	75.57 ± 7.49	76.08 ± 7.19	76.43 ± 6.98	76.47 ± 7.30	5.466	0.001
FPG (mmol/L)	5.30 ± 1.53	5.59 ± 1.73	5.76 ± 1.74	6.17 ± 2.14	67.125	0.000
TC (mmol/L)	4.63 ± 1.10	4.83 ± 1.22	4.96 ± 1.16	5.13 ± 1.13	55.456	0.000
TG (mmol/L)	0.80 ± 0.28	1.12 ± 0.37	1.46 ± 0.44	2.63 ± 1.61	1369.13	0.000
Family history of diabetes [n(%)]	68(4.2)	173(10.8)	315(19.4)	562(34.5)	583.673	0.000

*BMI* Body mass index, *WC* Waist circumference, *WHtR* Waist-to-height ratio, *SBP* Systolic blood pressure, *DBP* Diastolic blood pressure, *FPG* Fasting plasma glucose, *TC* Total cholesterol, *TG* Triglycerides

**Fig. 2:**
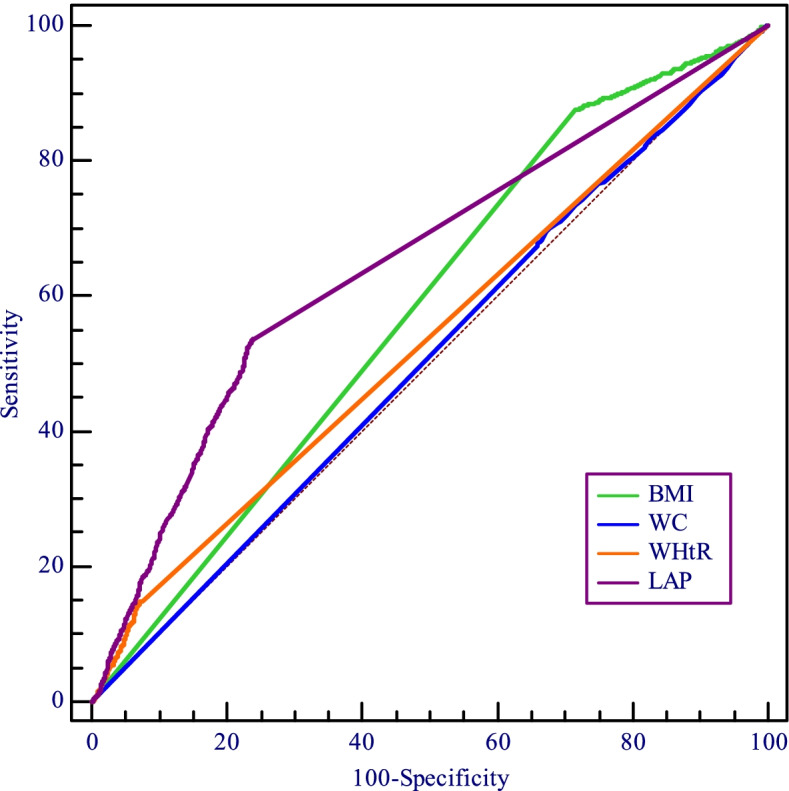
The receiver operating characteristic curve of different obesity indexes in predicting impaired fasting glucose and diabetes risk of the study population in country (Bengbu city, Anhui province, China), 2018

The results of ROC curve analysis were shown in Table 3. LAP (AUC = 0.650; 95% CI = 0.637 to 0.662) was a better indicator than BMI (AUC = 0.579; 95% CI = 0.566 to 0.592), WHtR (AUC = 0.537; 95% CI = 0.524 to 0.550), WC (AUC = 0.508; 95% CI = 0.495 to 0.521) to predict IFG and diabetes. The best cut-off value of LAP to predict IFG and diabetes was 33.54. It was found that LAP value could be used to diagnose IFG and diabetes risk (AUC = 0.650; *P* = 0.007). Sensitivity and specificity values for BMI, WC, WHtR and LAP were shown in Fig. 2.

**Table 3:** The comparison of obesity indexes in predicting impaired fasting glucose and diabetes risk of the study population in country (Bengbu city, Anhui province, China), 2018

Variables	Cut-off value	Sensitivity (%)	Specificity (%)	Youden index	AUC (95%CI)	*Z*	*P*-value^*^
BMI	25.14	48.86	64.96	0.138	0.579(0.566–0.592)	9.051	< 0.001
WC	83.5	64.38	48.66	0.131	0.508(0.495–0.521)	13.947	< 0.001
WHtR	0.53	51.53	59.85	0.114	0.537(0.524–0.550)	14.869	< 0.001
LAP	33.54	54.60	62.42	0.170	0.650(0.637–0.662)	-	-

^*^ Compared with that of LAP

The results of multinomial logistic regression analysis were conducted in Table 4. Subjects with the higher quartiles of LAP and family history were more likely to develop IFG and diabetes than those with the first quartile and non-family history. After controlling for confounders, the risk of IFG (adjusted OR: 2.735, 95% CI: 1.794–4.170) and diabetes (adjusted OR: 1.815, 95% CI: 1.297–2.541) significantly increased in subjects with the fourth quartile of LAP compared with the first quartile, meanwhile, increasing risk of diabetes (adjusted OR: 2.307, 95% CI: 1.787–2.978) was observed in family history participants compared with those non-family history participants.

**Table 4:** The logistic regression analysis of lipid accumulation product and family history of diabetes with impaired fasting glucose and diabetes of the study population in country (Bengbu city, Anhui province, China), 2018

Variables	OR (95% CI)^a^		OR (95% CI)^b^		OR (95% CI)^c^	
	IFG	Diabetes	IFG	Diabetes	IFG	Diabetes
Quartiles of LAP						
Q_1_	1.00(ref.)	1.00(ref.)	1.00(ref.)	1.00(ref.)	1.00(ref.)	1.00(ref.)
Q_2_	1.308 (0.987–1.733)	1.482^**^ (1.181–1.859)	1.357^*^ (1.021–1.803)	1.545^***^ (1.230–1.942)	1.327 (0.980–1.797)	1.421^**^ (1.115–1.810)
Q_3_	2.151^***^ (1.656–2.793)	1.680^***^ (1.342–2.103)	2.244^***^ (1.720–2.927)	1.778^***^ (1.417–2.232)	2.211^***^ (1.604–3.046)	1.526^**^ (1.172–1.988)
Q_4_	2.465^***^ (1.891–3.213)	2.416^***^ (1.939–3.009)	2.615^***^ (1.991–3.435)	2.629^***^ (2.097–3.295)	2.735^***^ (1.794–4.170)	1.815^**^ (1.297–2.541)
Family history of diabetes						
No	1.00(ref.)	1.00(ref.)	1.00(ref.)	1.00(ref.)	1.00(ref.)	1.00(ref.)
Yes	1.426^**^ (1.152–1.766)	1.939^***^ (1.634–2.302)	1.420^**^ (1.144–1.761)	1.935^***^ (1.629–2.298)	1.223 (0.895–1.672)	2.307^***^ (1.787–2.978)

Family history of diabetes was defined as one parent or both parents having diabetes

^a^ Unadjusted

^b^ Adjusted for age, sex, educational level, marital status, physical activity, smoking and drinking

^c^ Adjusted for age, sex, educational level, marital status, physical activity, smoking, drinking, SBP, DBP, BMI, WC, WHtR, TC and TG

^*^P < 0.05

^**^P < 0.01

^***^P < 0.001

The interaction analysis results were presented in Table 5. The adjusted OR of individuals with the highest LAP and family history of diabetes (2.820, 95%CI: 2.362 to 3.368) possessed the highest risk of getting IFG and diabetes as compared with the lowest LAP and non-family history subjects, the results of RERI (1.538, 95%CI: 0.167 to 3.612), AP (0.375, 95%CI: 0.118 to 0.631) and SI (1.980, 95%CI: 1.206 to 3.251) indicated a significant interaction effect of LAP and family history of diabetes on IFG and diabetes.

**Table 5:** The interaction analysis of lipid accumulation product with family history of diabetes and obesity on risk of impaired fasting glucose and diabetes of the study population in country (Bengbu city, Anhui province, China), 2018

Variables		case/total	OR^b^ (95% CI)	Measures of interaction		
				RERI	AP	SI
LAP^a^	Family history of diabetes			1.538^*^ (0.167–3.612)	0.375^*^ (0.118–0.631)	1.980^*^ (1.206–3.251)
Low	No	3453/3775	1.00(ref.)			
Low	Yes	322/3775	1.898^***^ (1.466–2.459)			
High	No	1896/2692	1.566^***^ (1.341–1.830)			
High	Yes	796/2692	2.820^***^ (2.362–3.368)			
LAP^a^	General obesity			1.221^#^ (-0.183–2.356)	0.245^#^ (-0.010–0.476)	1.453^#^ (0.904–2.357)
Low	No	3555/3775	1.00(ref.)			
Low	Yes	220/3775	1.320 (0.950–1.833)			
High	No	2064/2692	1.732^***^ (1.493–2.009)			
High	Yes	628/2692	2.202^***^ (1.812–2.675)			
LAP^a^	Abdominal obesity			1.492^*^ (0.087–3.723)	0.413^*^ (0.014–0.756)	1.824^#^ (0.873–3.526)
Low	No	2947/3775	1.00(ref.)			
Low	Yes	828/3775	1.130 (0.925–1.382)			
High	No	854/2692	1.469^***^ (1.197–1.803)			
High	Yes	1838/2692	1.997^***^ (1.722–2.315)			

^a^ Grouped by the cut-off value (33.54) in Table 3

^b^ Adjusted for age, sex, educational level, marital status, physical activity, smoking, drinking, SBP, DBP, TC and TG

^*^ *P* < 0.05

^#^ *P* > 0.05

However, no statistically significant interaction effects were found between LAP and general obesity, RERI was 1.221 (95%CI: -0.183 to 2.356), AP was 0.245 (95%CI: -0.010 to 0.476) and SI was 1.453 (95%CI: 0.904 to 2.357). Moreover, compared to individuals with low-LAP and non-abdominal obesity, the adjusted OR of subjects with high-LAP and abdominal obesity was 1.997 (95%CI: 1.722 to 2.315), however, the results of RERI (1.492, 95%CI: 0.087 to 3.723), AP (0.413, 95%CI: 0.014 to 0.756) indicated a significant interaction of LAP and abdominal obesity, but the result of SI (1.824, 95%CI: 0.873 to 3.526) did not.

## Discussion

With the high prevalence of overweight and obesity, unhealthy dietary habits and sedentary lifestyles, IFG and diabetes is increasingly common among community residents, and has become an important public health issue [41, 42]. The International Diabetes Federation has predicted that the number of individuals with diabetes will increase to 380 million in 2025 and 439 million in 2030 [43, 44]. The prevalence of diabetes and IFG was 14.33% and 9.37% in this population- based survey, with other studies reported in China, the prevalence of diabetes was as high as 30.6% in Heilongjiang and 17.9% in Jilin, whereas in some provinces the prevalence was as low as 3.6% in Yunnan and 5.9% in Zhejiang and 12.5% in Hunan [45]. Participants living in Jilin and Liaoning Province showed a relatively high prevalence (11.8% and 16.5%, respectively) of IFG compared to our study [46, 47], we found that the prevalence in the whole country is imbalance in geographic areas, with a vast territory, China has a tremendous difference among different regions, the levels of economic development and lifestyles differences may influence the epidemic of IFG and diabetes.

In most population-based studies, BMI, WC, WHtR are the most common obesity indexes, but there are some limitations in using these indicators to diagnose obesity. Firstly, BMI has inherent inability to distinguish subcutaneous fat and visceral fat. Secondly, BMI does not characterize body fat distribution, which was known as a determinant metabolic risk, in this aspect, WC and WHtR might better represent visceral fat. Finally, although WC and WHtR can represent central obesity, they can not show the excess body fat in circulating blood. However, a new obesity index that can predict body fat simply and effectively is urgently needed. After the first introduce by Kahn [20] showed that LAP is an index of excessive lipid accumulation and perform better than BMI for recognizing diabetes risk, several studies revealed the association between LAP and cardiovascular risk over the past years [48, 49]. LAP, a combination of WC and TG, can reflect visceral fat excess and has theoretical basis to evaluate visceral obesity [50]. In our study, we analyzed data from a community health management project, tried to investigate the relationship between LAP and IFG, diabetes, and compared LAP with BMI, WC, WHtR for diabetes diagnostic accuracy. The prevalence of IFG and diabetes gradually increased across LAP quartiles, the values in the fourth quartile of LAP were dramatically higher than in the first quartile (12.6% vs. 5.9%, 20.8% vs. 8.9%, respectively), and this conclusion was consistent with other similar studies in China. A cross-sectional study in Beijing showed that an elevated level of LAP was closely linked to an increased risk of diabetes in elderly people [51], and a survey in Anhui province confirmed that there is a positive correlation between LAP and IFG [52].

The results showed that the AUC of LAP (0.650) for predicting IFG and diabetes was higher than that of BMI (0.579), WHtR (0.537) and WC (0.508), indicating that LAP had more power for predicting IFG and diabetes compared with common obesity indexes. In addition, the identified cutoff value of LAP was 33.54 (sensitivity, 54.6%; specificity, 62.42%) in study population, a number of studies have found similar results. A study in northeastern Brail showed that the cutoff value of LAP index to present a higher chance of cardiovascular risk was 37.9 [12], an investigation conducted among Urumqi in China found that LAP was better than BMI to predict cardiovascular risk and the predictive accuracy was 38.41 in 215,651 adults [31]. Meanwhile, in southern Taiwan, China, Chiang et al. [53] evaluated that the optimal cutoff value for the LAP index to predict diabetes was 28.4. There is still controversy about the optimal cutoff value of LAP to present cardiovascular risk, further studies with a large sample size stratified into age, sex and nutritional status categories (obesity or normal) are needed to investigate.

The interaction analysis in this study indicated that there was a significant interaction between LAP and family history of diabetes on the risk of IFG and diabetes, the etiology of diabetes is not well defined, but family history of diabetes has been considered a reflection of both genetic and environmental effects [54], and individuals with family history of diabetes are two to three times more likely to develop diabetes than those without family history individuals [55]. Similar reports reported the significant correlation between family history and risk of diabetes and IFG, a cross-sectional study in Sweden showed that family history of diabetes had an interactive influence on IFG in females [56]. An investigation conducted by Ustulin et al. [57] proved a relevant significant association between family history of diabetes and risk of diabetes in middle-aged and elderly person. However, no significant interaction between LAP and general obesity was observed in study population. Previous studies have explored the interaction effect between LAP and abdominal obesity on risk of diabetes [52, 58], but our result seem to be inconsistent. The mechanisms underlying the interaction between LAP and obesity are complex. Firstly, the mechanisms of obesity-induced diabetes include adipocytes increase, insulin resistance, inflammatory cytokines and relevant adipokines secretion [52]. Secondly, the mechanisms of diabetes increase can be activated by visceral fat [52]. Thirdly, the participants in our study were all middle-aged and elderly, the interaction need to be further explored in younger groups. The results of RERI, AP indicated a significant interaction of LAP and abdominal obesity, but the result of SI did not. Abdominal obesity may result in increased blood glucose through some unknown mechanisms. So far, there are few surveys exploring the interaction of risk factors on IFG and diabetes risk, and the interactive mechanisms between LAP and other factors needs to be studied in the future.

There are some limitations in our study. First, as a cross-sectional study, the causal association between LAP and IFG, diabetes can not be determined. Secondly, we did not identify different cutoff values for LAP according to sex, age and nutritional status categories. Thirdly, the population of our study can not fully represent the general population in the center of China. Finally, the enrolled individuals in this study were all middle-aged and elderly. So we suggest that additional longitudinal studies should evaluate in different sex and age groups, given that the association between LAP and IFG, diabetes risk is well-established.

## Conclusion

In conclusion, LAP significantly associated with the IFG and diabetes risk and performed better than other obesity indices. Furthermore, LAP with family history of diabetes may have an interactive effect on the development of IFG and diabetes. Further studies should pay more attention to the gender and age differences of LAP and the mechanisms of interactive effect on IFG and diabetes risk.

## Supplementary Information

**Additional file 1. **

## Data Availability

Data available within the article or its supplementary materials.

## Abbreviations

LAP: Lipid accumulation product
IFG: Impaired fasting glucose
BMI: Body mass index
WC: Waist circumference
WHtR: Waist to height ratio
ROC: Receiver operating characteristic
RERI: Relative excess risk of interaction
AP: Attributable proportion due to interaction
SI: Synergy index
SBP: Systolic blood pressure
DBP: Diastolic blood pressure
FPG: Fasting plasma glucose
TC: Total cholesterol
TG: Triglycerides
OR: Odds ratio
SD: Standard deviation
CIs: Confidence interval
